# The spectacle of feminism and machismo in two Peruvian cumbia singers: Marisol and Tony Rosado

**DOI:** 10.3389/fsoc.2022.978403

**Published:** 2023-01-09

**Authors:** Edgar Gutiérrez-Gómez, Sonia Beatriz Munaris-Parco

**Affiliations:** ^1^Escuela de Administración de Turismo Sostenible y Hotelería, Universidad Nacional Autónoma de Huanta, Huanta, Peru; ^2^Área de Ciencias Sociales, Institución Educativa Pública “Nuestra Señora de Fátima”, Ayacucho, Peru

**Keywords:** Quechua, cinema, Andes, identity, sexism

## Abstract

The objective of this research is to analyze and interpret the entertainment section of the most important media in Peru, concentrated on two private companies: Grupo La República and Grupo El Comercio, with great journalistic dominance of national information. The entertainment section is as old as the foundation of the newspapers themselves. The method of analysis with qualitative documents has allowed us to reach the conclusion that the fight for gender equality promoted by the government is a spectacle for the national press. The entertainment section of the Peruvian press has exposed the private lives of representative characters such as the singers Marisol, “La Faraona de la Cumbia” and Tony Rosado, “El Ruiseñor de la Cumbia”, from there the differences in feminism tolerated in Marisol and machismo censored in Rosado are popularized. It is concluded that the exposure of the struggle for gender equality is entertainment news where machismo and feminism are underhandedly justified with the parameter established with these public figures, that is, Peruvian cumbia singer–songwriters, and that offers evidence of the tolerance to the feminine voice that incites machismo, justifies the mistreatment of men and makes the male complaint a synonym of cowardice.

## Introduction

Governments, in different parts of the world, deal with and legislate the defense of gender equality, especially women's rights, due to the long history of paternal machismo in the different manifestations of society. The evolutionary development of humanity was adverse for women in their struggle to vindicate their gender in social standards of rational acceptance. Incrementation to this is the proliferation of Protestant churches that encourage machismo in the popular sectors: “75% of Peruvians are Catholics, 14% Evangelicals, 5% of other religions and 6% agnostics or atheists. The trend is declining” (Torres, 2018). These congregations interpret the Bible literally through their evangelical pastors who personify the earthly divinity for their parishioners.

The media considered the “fourth estate,” plays a decisive role in the education and re-education of the population. In Peru, most of them are divided into two groups: El Comercio and La República, being almost the only owners of information and disinformation, which have an editorial line according to the great financial interests. Access to information is exclusive to these two large business groups since they have printed newspapers, television or video programs, and radio programs for each sector of society, which are accessed according to the culture and place they occupy in Peru's geographic space. These media, concentrated on two business owners, have special entertainment or show business sections dedicated to frivolity in the daily news.

The essence of the research of this article is to analyze feminism and machismo in entertainment and show business news. It is necessary to analyze and expose the underhanded information of the national problem of machismo and feminism in the media in the entertainment section: “machista societies are where most men abandon children, where taking care of children is considered a woman's issue, and Latin American society is enormously machista and religious societies are conservative and patriarchal” (Bel, 2021). Iris Marisol MuguerzaMego nicknamed in the artistic world “Marisol, la faraona de la cumbia (the Female Pharaoh of cumbia)” and Victor Agustin Rosado Adana, artistically nicknamed “Tony Rosado, elruiseñor de la cumbia (the Nightingale of cumbia)” expose crude apologies of machismo and feminism in Peru, which should be worrisome news, but is spread in the entertainment section of the mass media. Peruvian society has only two corporate media segmented for each sector stratified as A, B, C, D, and E; therefore, access to news is restricted and invaded by the so-called chicha (peruvianism press show) press: “characterized especially by its vulgar style of exploitation of the female sex, attacks and hurtful adjectives to public figures, the elderly, the disabled and everything that may be new, useful, impactful or of human interest” (Cappellini, 2004). The power of journalistic information influences the popular sectors due to the lack of heterogeneity, plurality, and veracity of the entertainment section.

The public user considers feminism and machismo are part of the spectacle in Peru, which is part of the gossip of everyday life. As feminism and machismo exalted by the “Faraona de la cumbia” and the “Ruiseñor de la cumbia” is a spectacle in their different presentations, it is entertainment news that is not considered a social danger for their followers who consume it as frivolous news; as such, machismo and feminism extremes by the singer–songwriters feed the mistreatment against women and men. The Peruvian state established the Law of Equal Opportunities between Women and Men No. 28983 in 2007, which is the theoretical framework that supports this research, endorsed with the founding of the Ministry of Women and Vulnerable Populations in 1996, and whose mission is to prevent, eradicate and punish gender violence in its different manifestations. This research aims to demonstrate the little importance given by the media, divided into two monopolized business groups in Peru, to the struggles for gender equality, placing in the entertainment section the important news about male and female mistreatment in Peruvian singer–songwriters.

## Scenarios of machismo and feminism in Peruvian cumbia

Composers, performers, and songwriters worldwide subsist on their audiences who buy tickets to their concerts and national and international tours. Each musical genre has specific followers and audiences to whom the content of their inspiring performances is directed. The triumph of provincial artists is not enough outside the metropolis of Lima's centralism that marginalizes the provinces; thus, artists have to migrate to the capital of Peru to spread their music. In the words of Marisol: “for me it has been a little difficult to leave my town since all my life I have lived in Chiclayo and getting used to Lima, to another type of environment, it is very difficult,” added the singer in tears in “El gran show” (The Great Show) (Comercio, 2015).

Extreme feminist expressions against men are accepted as normal in Peruvian society, especially by the media in their entertainment sections, and chanted in unison as: “with Marisol we have come together in this show at the request of the public, thanks to DEA Promotora. They are going to have traditional Creole, black and cumbia music... let that men get ready,” said Eva Ayllón (Comercio, 2016). These expressions expressed against men in the show programs that swarm the Peruvian press should be questioned by the authorities who defend gender equality, but these expressions on the part of the singers are accepted as a defensive standard.

The male version that bursts into macho manly actions and laments in his artistic presentations is that of the “Ruiseñor de la cumbia,” constantly questioned for uttering macho phrases against women, which have the same morbidness as those of the “Faraona de la cumbia,” but she is tolerated in all the media, entertainment section. Both characters express feelings of their personal lives, evidencing their family failures. Rosado confesses: “‘I arrived home quietly and found her with my neighbor. That is where my story began'. This bitter experience would have formed his character on stage, which emits bitter and violent comments against women” (Comercio, 2019c). The public that follows the artistic presentations identifies with the character and chants his songs while drinking liquor, crying loudly with the song, and expressing their comments on each stage where he performs. The artistic presentations are every weekend in different parts of the Peruvian territory with a captive audience that fills the artistic stages set up for this purpose.

At a national level, in every town, there are countless festivities where artistic presentations are valued, which are propitious spaces for the presentations of Tony Rosado and Marisol. The captive audience of the artists always requires them to see them in the musical presentations they attend: “after the success of ‘Nacida para triunfar' (Born to triumph), a biographical TV series of singer Sonia Morales, we had to look for a new story equally or more exciting. Marisol was in fashion, and her life was worthy of being brought to the screen” (Águila, 2021). In Peru, there are holidays or non-working days calendared as mostly religious holidays; also, from time to time, the government in power ingratiates itself with the state worker and decrees some weekends or early in the weeks as national holidays to promote domestic tourism.

The life of the artists referred to, then, is no stranger to miniseries and special attention in Peru. Fans of Tony Rosado's songs and music do the same by requesting his presence at important events: “fans dissatisfied for not including Tony Rosado's songs in the *playlist* of the Pan American Games” (República, 2019e). In the same way, the show business section of the Peruvian press has more tuning in television and radio audiences and readership of written circulation focused on the artists, subject of this investigative study: “Marisol, The Female Pharaoh of Cumbia, and Tony Rosado will return to deliver the best of their songs in a show to be presented on the Central Highway” (RPP., 2011). All media outlets have a daily *rating* section on the positioning of the show business section. In one case, a hostess was: “qualified as sexist, retrograde. Magaly TV La Firme (The True One) had 10.5 points in Lima and 9.8 in Lima +6 ciudades (+6 cities), being in sixth place. The morning show +Espectáculos (+Entertainment) had 4.5 points in Lima and 4.1 in Lima +6 ciudades” (Perú, 2022).

### Political parties in the macho and feminist campaign

The political parties personified by their occasional leaders on duty, feed machismo and feminism knowing that in Peru they: “establish measures to prevent situations of political harassment, such as those related to dissemination and awareness campaigns, conducting studies and research that help to learn more about the situation of women, in particular, from an intersectional view” (Freidenberg and Gilas, 2022; p. 9). For their part, the leaders in electoral competition identify themselves with the cumbiambera music to promote their candidacy in festive scenarios, distribute liquor and finance popular parties:

A few days ago, the municipality of Tarapoto announced the list of artists who will be invited for the activities of the 237 years of the Spanish foundation of Tarapoto. Tony Rosado was going to perform on Tuesday, August 20. The promoter who was going to hire him said in a statement that he will not be included in the event, for justifying the mistreatment of women (Anteparra, 2019).

At the national level, local municipalities have funds and offices dedicated to the promotion of popular culture in order to identify and promote the indigenous culture of the towns. In these artistic presentations, due to the effects of alcohol, they vent their sorrows and frustrations that they could not express in sobriety. The observations and criticisms of the politicians when they assume power or have some position are delimited by the offenses that the singers can offer. Criticisms with adjectives, if they come from a woman against men, as in the case of the “Faraona de la cumbia,” are considered contemplative; on the contrary, if it is a man who pronounces himself with adjectives against women, he is condemned and slammed in the same media, in the entertainment section, as reported here:

This man is disgusting. I can't think that he would be hired again and that he would leave the woman like that. We have to remind this asshole that he was born of a woman, right? Why so much anger against women, he must have been a cuckold, right? What is one's fault if he does badly in love and then comes out saying bestiality and a half,” said the former minister (República, 2019f).

The comment of the former Minister of Women is adjectival and pejorative; as such, it continues to feed extreme feminism to have indicated a “cuckold” for the action of the infidelity of a woman that the former minister minimizes. Justifying the “cuckold” of a man because of a woman's infidelity seems to be a normal situation. In an opposite scenario, if a former minister were to publicly declare as the former minister did, the consequences would be disastrous for men; however, the macho opinions of women are contemplated and even accepted. The vulgar expressions emitted by the singers mentioned in this article are received with acclaim by the attendants to their events, and politicians always have an active participation with the singer–songwriters who are popular:

Tony Rosado had no qualms in assuring that the burgomaster told him that he hired him to say the characteristic swear word that is part of his popular song. “Mr. Mayor has told me ‘I have hired you to say mother f^***^er'. I have it recorded there just in case,” he said (República, 2019i).

Rosado's insults against someone's mother in his presentations are cheered by the audience identifies them as being for women who have already forgotten their past marital relationship. Rosado's musical presentations have long introductions that allude to an aggressive machismo, such as: “...I've already forgotten you, I've already forgotten you, I'll mother f^***^er” (Producciones, 2017; pt. 1:51). Attendees eagerly await their comments and chant in fascination; hundreds of young people and adults attend their presentations, and no one is uncomfortable with their expressions. This duo of musicians, which is dedicated in Peru to performing shows in favor of machismo and feminism, both vulgar and extreme, is approved by political organizations and entities that promote and control cultural diffusion. The essence of the controversy, which goes unnoticed, is the demonstrations for and against Marisol:

After Tony Rosado was accused of inciting violence against women through the words and attitudes in his concerts, through social networks an event was organized against the singer Marisol called “March for Marisol to stop denigrating men.” (República, 2019g).

Peruvian society struggles for the defense of gender equality: “one of the challenges of Peruvian feminism is how to respond to the context of social, political and economic changes in Peruvian society. A society without political parties, but rather electoral fronts” (Silva and Cabrejo, 2014; p. 643). With a Ministry of Women and Vulnerable Populations, this has tacitly allowed the denigration of men and has become common practice in the show media, as the political practice itself in the Congress of the Republic, and political campaigns at the national level demonstrate: “several cases of gender violence in Congress paraded through the Ethics Commission, chaired by Karol Paredes (Acción Popular). However, the complaints were shelved” (García, 2022). Men must constantly measure their expressions in political debates and the media, while women express themselves with total immunity, as happened in a TV show: “Gisela Valcárcel intervened to flatter the singer and dancer. “Please, I have to say it. That was wonderful, careful if I see you [man], I break your snout. You're a great one,” she said. “You have to conclude that,” Pamela replied” (Ojo, 2021).

Important public figures on Peruvian television approve of the expressions of women against men with total ease, and the Ministry of Women does not speak out about it. Marisol, such as Tony Rosado against women, expresses vulgar comments toward men in her concerts in the presence of hundreds of attendees who listen to her song: “La escobita” [The Little Broom]: “... but, when I see you, I'll kick your... you know...” (XefabianX, 2020; pt. 3:02). The expressions that Rosado utters are the same in her concerts which were censored with polemic declarations of different institutional entities of the state, influential personalities in Peruvian politics, while with Marisol the attitudes are contemplative and go unnoticed.

### Research on the macho attitudes of singer–songwriter Tony Rosado

In Peru, from time to time, extreme macho and feminist attitudes have manifested that end up as anecdotes of journalistic spaces in shows, cases referred to as:

President Martin Vizcarra made reference to some of the victims of femicide such as Elsa Salas De La Cruz, Gaby Vásquez Gómez, Micaela LamanAcho, Gloria Pacheco Yucra, Mary Huayllapuma Lima, Yudith Solis Leandro, Lucinda Vásquez Sánchez, EyviAgreda Marchena, Olga Aponte Mariño, Juanita Mendoza Alva, Mayra Pérez Gilberti, Dilma Suarez Jiménez, Estefany Taboada Mendoza and all those anonymous victims of femicide (Prensa, 2018).

Also, some politicians had past events tinged with machismo and feminism, as in the case of the president of the Women's Commission of the Peruvian Congress: “Maritza García said that women 'sometimes without reason give men the opportunity' to commit femicides” (De La Quintana, 2017). These are some cases of the Peruvian political scenario, which are the news of spectacle in schedules not suitable for minors. Regarding Tony Rosado “the Ombudsman, Walter Gutiérrez, asked the Prosecutor's Office to open an investigation against him for apologizing for aggression against women” (Comercio, 2019b). It is an opinion against the macho attitudes of Rosado, who is investigated for his comments in his public presentations, taking into account that hundreds of people attend his concerts of their own free will and pay a certain amount of money for the presentations tickets:

“Expressions of singer Tony Rosado are absolutely despicable and unacceptable for inciting violence against women.” “@FiscaliaPeru must initiate investigation immediately. Apology (Art. 316 of the Penal Code) to femicide is a crime and as a society we cannot allow it,” Gutiérrez wrote on his Twitter account (Comercio, 2019a).

Demonstrations against Rosado's expressions were issued after his constant presentations nationwide. In all his presentations, there was no lack of insults against women's mothers and incitement of hatred toward them for their unfaithful behavior by going with other men. The musical solidarity is accompanied by praise from other artists who consider him the best interpreter of Peruvian cumbia, as in the case of DeyvisOrosco, who declared: “the interpreter of ‘Gotitas de lluvia' [Tiny drops of rain] described Tony Rosado as a ‘Maestro'. “For me, Tony Rosado will always be a Maestro” (República, 2019c).

Opinions about the controversial expressions with a macho sense of Rosado are divided as the news says: “in the video not only Tony Rosado is observed insulting women and the driver but also referring to rape, in a light way. The name of the ‘Ruiseñor de la cumbia' is trending on Twitter” (República, 2019b). In one of the many presentations, Tony expresses: “there is a lady who appeared on television (Magaly Medina) to tell me that I am a criminal, who have I killed, who have I raped? No one. The only one I have raped is my wife and that's why they call me a delinquent” (Perú21, 2019). As evidenced in the publications of newspapers of national circulation in Peru, the most pressing problem of gender violence is entertainment news, without any importance for the authorities who should prevent violent expressions against men and women in Peru.

There are a series of shows that broadcast “showbiz” content, so-called by the same media. Tony Rosado was at the center of the news in relation to machismo in a show business program where people from his close environment intervened: “it was Tony Rosado's wife who asked him to show his face in order to apologize to all the women who have felt offended by the words he said” (República, 2019d). After receiving a series of remarks about his behavior in his musical presentations, Rosado changes his speech with other words that still have the same effect, but with special care, he modifies his vocabulary in all his presentations. The reporters of the media, and show business section, made constant follow-ups to all his presentations. The Tony Rosado phenomenon was news in the entertainment section, taking into account the problematic nature of the denunciations for his aggressive macho manifestations:

He thinks that, if the girls wear miniskirts, they have to be raped. If she is with cleavage, you have to stick your hand in. You are a disgrace, you humiliate women. We have to listen to you so you don't kill us. He's sick and tricky, that's what this guy is (República, 2019a).

The observations and questioning of Rosado's statements follow the same thread of offensive and pejorative language. In response to these remarks, the singer–songwriter continues to use sexist language with a subtle vocabulary of irony, such as “distinguished lady,” instead of a mother's insult.

### Artistic stages of Marisol, *the Pharaoh of cumbia*

The lack of balance in research and judgments of relevance on the human gender makes machismo and feminism evident in Peru. Popular manifestations should be neutral for both genders, but popular justice and legislations are contemplative for one of them; in this case, expressions of violent feminism are allowed in women: “Marisol is in the eye of the storm after giving her opinion about Tony Rosado and his controversial concerts. Now, users of social networks have called for a march against him, and she was compared to the ‘Ruiseñor de la cumbia”' (República, 2019h). This demonstration was quite a spectacle and went unnoticed because men were seen as weak before the feminist sector of society, marching in defense of their rights to be offended with their songs that, yes, have much to do with Tony Rosado: “Canalla” [Scoundrel], “La escobita” [Little Broom], and “Yo lo quería” [I loved him] are some of the songs where Marisol used certain passages of her love life as inspiration” (República, 2020). The music she plays in her concerts shows her private life and is aired with expressions of anger and pain, similar to those of the audience who identify with the lyrics of the singer–songwriter's music.

In constant showbiz news about the artistic life of Marisol and Tony Rosado, she balances her repudiation of him with statements about her personal life: “I do not rule out anything, nor do I say that I will not drink from this water. I leave everything in God's hands. There are many women who forgive for the sake of their children, but it must be for the sake of love. The truth, I do not deny it. I still love my husband, but that will pass” (Redacción RPP, 2012). The expressions referred to are notorious in the singer's followers; as the women of the popular sectors of Peru say, it is a way of justifying the negative actions of men; however, what the singer emits in her presentations is contrary to repudiation to them. In the history of Peruvian music of the cumbia genre, there is no evidence of marked manifestations of machismo and feminism, as noted in this part of an article:

Among the first groups that ventured into the tropical environment, “andinizing” the melody, were the Sanders de Ñaña, Los Orientales de Paramonga, Los Diablos Rojos de Marino Valencia and Grupo Celeste. It was the latter that had as one of its vocalists Alfredo Escalante Quispe, “Chacal”, one of the first singers who gave the “ahuaynado” tone to cumbia (Bailon, 2013, p. 54).

Cumbia music is linked to the popular expressions of the Peruvian highlands and provinces, where it shows the manifestations of machismo from ancestral times, as well as the way of living in the field of agriculture. The huayno musical genre, which is purely provincial, reflects the spectrum of suffering in the countryside, in the transformation of the land and planting, only in times of rain once a year. The metropolis society, which is the capital of Lima, looks askance at the provincials, who do not adapt to the capital's social class that has social interrelations with foreigners; hence, the social movement of migration from the countryside to the city:

Marisol, La Faraona de la Cumbia, enters the select circuit of “Cumbia VIP” and will take her “strong woman image” next Thursday, February 19 to the stage of a crowded nightclub in Lince, in a concert dedicated to all the “pitucas” [posh girls] in which she promises to crucify all the infidels with her hits “30 segundos” [30 s] and “Canalla” [Scoundrel], besides being the first time that the singer performs in a nightclub of another purchasing power level (Redacción RPP, 2009).

Marisol, as a female public figure who summons and acclaims thousands of fans nationwide, distorts the image of women and men in Peru with her musical content of macho and feminist expression. The musical classification of genres by social class is another negative factor in the struggle for gender equality in Peru. Women's actions and manifestations are neglected by politicians and legislative decision-makers. In political life, the power quotas established in the legislation feed the participation of women and young people as an obligation, without previous evaluation of their competencies for a specific position; many times, they only participate as a complement to fulfill the requirements of the gender quota in the political parties of Peru.

## Conclusion

In Peru, the media glorify informative content in accordance with the editorial line of their economic interests. Political scandals linked to the private lives of their protagonists are the economic income of media companies; they are the ideal space for the entertainment section of the media: “basically, the media privilege certain ways of constructing the news, to highlight them over others” (Turpo Gebera, 2020; p. 469). These media overlap issues of social importance with irrelevant information in entertainment news that complements the current political issues. The private lives of show business personalities have a higher profile in the society that consumes this type of news on a daily basis. The media's coverage of Marisol and Tony Rosado in the entertainment section is constant, which encourages machismo and feminism in Peru.

The vindicatory struggles for gender equality are belittled by the media in the entertainment section, as indicated in this article; since they violate the norm: “according to Article 40 of the Radio and Television Law, during 6:00 and 22:00 h, programming must avoid violent, obscene or other contents that may affect the inherent values of children, adolescents and the family” (El peruano, 2022).

The radio stations that, at a national level, broadcast Peruvian cumbia also feed the machismo and feminism of Tony and Marisol's expressions, and to this are added the macho and feminist humor programs, such as “Qumbias y Risas[Cumbias and Laughs] with Edwin Sierra and Oscar Del Río as hosts and El Show de las Mamis [The Mommies Show] with Chris Alegría as host” (Radio nueva Q, 2022). In these programs with assured ratings, the show of feminism and machismo with the music of Tony Rosado and Marisol is in tune with the popular sector of Peruvian society. Their music is broadcasted daily with humor in Cumbiaradios. To these stations, Radio Nueva Q [New Q Radio] and the program “El Super Show de la Karibeña” [The SuperShow of the Caribean] are added: “From Monday to Saturday from 6 to 9 a.m., with Kike Suero and Miguel Moreno, who are the vaccine against boredom on radio La Karibeña ¡SíSuena!” [Caribean Radio, Yes, it sounds!] (Radio Karibeña., 2022). These are some radio programs that spread feminist and macho jokes among a popular audience.

A broader political space is required to discuss and legislate on artistic presentations and, above all, the control of information that discredits formal education in Peru. The consumption of information by the power groups leaves minimal space for other journalistic companies that broadcast culture and education as an independent press. On the other hand, analyzing the message of the artists referred to in this study is the task of political decision-makers, who take advantage of the popular fame of the singer–songwriters, while neglecting the laws that they themselves enacted.

The limitations of this research are due to the excessive broadcasting of entertainment programs in different media and scattered schedules, managed by the two large journalistic groups that do not respect the laws of content control, which require a sustained statistical treatment.

## Author contributions

EG-G developed and directed the research project and led the writing of this manuscript. He did the search and analysis of the literature on this topic and also contributed to the analysis and interpretation of data and the revision of the English version and writing in Frontiers format. SM-P drafting and review of the draft and original. All authors contributed to the article and approved the submitted version.
